# A lightweight YOLOv11-based framework for small steel defect detection with a newly enhanced feature fusion module

**DOI:** 10.1038/s41598-025-16619-9

**Published:** 2025-10-02

**Authors:** Yongyao Wang, Haiyang Sun, Kai Luo, Quanfu Zhu, Haofei Li, Yuyang Sun, Zhenjie Wu, Gang Wang

**Affiliations:** 1 College of Electrical and Information Engineering, Beihua University, JiLin, 132000 JiLin China; 2 College of Electronic and Information Engineering, Changchun University, Changchun, 130022 JiLin China

**Keywords:** yolo 11, Ghost convolutions, Multi-dimensional-fusion neck, Attention concat, Virtual fusion head, Computer science, Aerospace engineering, Civil engineering

## Abstract

In order to address the challenges of deployment difficulties and low small-object detection efficiency in current deep learning-based defect detection models on terminal devices with limited computational capacity, this paper proposes a lightweight steel surface defect detection model, Pyramid-based Small-target Fusion YOLO (PSF-YOLO), based on an improved YOLOv11n object detection framework. The model employs a low-parameter Ghost convolution (GhostConv) to substantially reduce the required computational resources. Additionally, the traditional feature pyramid network structure is replaced with a Multi-Dimensional-Fusion neck (MDF-Neck) to enhance small-object perception and reduce the number of model parameters. Moreover, to achieve multi-dimensional integration in the neck, a Virtual Fusion Head is utilized, and the design of an Attention Concat module further improves target feature extraction, thereby significantly enhancing overall detection performance. Experimental results on the GC10-DET+ dataset demonstrate that PSF-YOLO reduces model parameters by 25% while achieving improvements of 3.2% and 3.3% in $mAP_{50}$ and $mAP_{50-95}$, respectively, compared to the baseline model. This approach offers valuable insights and practical applicability for deploying defect detection models on terminal devices with limited computational resources.

## Introduction

Since the advent of the Internet era, the significant increase in computing power has driven rapid advancements in computer technologies such as artificial intelligence and deep learning, which are increasingly applied in the industrial sector. These technologies have facilitated the transformation of traditional manufacturing and engineering maintenance toward digitalization and intelligence^1–3^. One important application of artificial intelligence in engineering is defect detection technology. In metal production and application, object detection techniques have demonstrated considerable advantages in identifying surface defects. As metals become more widely used, applying object detection in metal defect inspection has significantly improved efficiency and reduced the incidence of false or missed detections. However, in practice, defect detection models often struggle with low accuracy for small targets, diminishing user confidence and resulting in considerable resource wastage. Furthermore, these models typically contain a large number of parameters, which poses significant challenges for deployment.

Steel surface defect detection methods can be broadly classified into three categories: traditional defect detection, machine vision-based defect analysis, and deep learning-based defect detection^4,5^. Traditional methods—such as visual inspection, magnetic particle testing, penetrant testing, ultrasonic testing, and radiographic testing are prevalent in industrial production but generally suffer from low efficiency, heavy reliance on human expertise, high cost, and inadequate real-time capability, making them unsuitable for the high precision and efficiency demands of modern steel production^6^. In contrast, machine vision-based defect analysis reduces dependence on human intervention by employing high-resolution cameras for image capture, followed by image pre-processing (e.g., denoising, enhancement, and edge detection) and traditional machine learning techniques (e.g., support vector machines and BP neural networks) for defect classification and recognition^7^. However, these methods often exhibit limited adaptability to complex backgrounds and poor generalization capabilities.

Deep learning-based defect detection methods can effectively extract features from input data, enabling accurate recognition in complex backgrounds. Moreover, these models do not require manual parameter adjustments or retraining for different scenarios, thereby enhancing their transferability, flexibility, and generalizability. Recent research has improved detection speed while maintaining accuracy through neural network pruning and optimization of the Faster R-CNN model structure^8^. Additionally, some studies have replaced the VGG16 backbone of the SSD model with a FasterNet network constructed using PConv to reduce computational complexity^9^. In medical image segmentation, integrating U-Net with Transformer modules has been shown to enhance segmentation and prediction accuracy^10^. Although these approaches have significantly improved model accuracy and precision, high-precision models tend to have complex structures that are difficult to deploy in practical applications. Conversely, lightweight models often sacrifice generalization and robustness, leading to lower accuracy and suboptimal detection performance in real-world scenarios. Therefore, a novel algorithm is urgently needed—one that balances a lightweight design and low parameter count with high detection accuracy.

1. We propose a novel Multi-Dimensional-Fusion Neck (MDF-Neck) that introduces dense cross-scale connectivity and adaptive weighting across the P1–P4 feature layers. This design enhances multi-scale feature integration and improves the model’s robustness to steel defects of varying sizes and complex backgrounds.
2. A dual mechanism is integrated into the neck: (a) the Virtual Fusion Head adaptively enhances potential small-defect regions through resolution-aware pooling and alignment, and (b) the Attention Concat module guides feature aggregation via lightweight spatial-channel attention. This synergy significantly improves the model’s sensitivity to low-contrast, small-scale defects.
3. GhostConv replaces conventional convolutions in the backbone, leveraging low-cost linear transformations to generate redundant features. This reduces parameters by 25% while maintaining or improving detection accuracy, enabling real-time deployment on edge devices in industrial settings.
4. A domain-aligned enhancement of the GC10-DET dataset is performed, including small-defect-aware data augmentation, class balancing, and re-annotation. This adaptation addresses annotation scarcity and improves training stability, allowing better real-world generalization for steel surface defect detection.

## Related work

### Surface defect detection

With the widespread adoption of deep learning in both industrial production and everyday life, researchers have increasingly focused on advancing defect detection technologies. For instance, some studies have optimized the spatial pyramid pooling in YOLOv5 by replacing the SPPF module with SinSPPF, thereby reducing computational complexity and accelerating detection speed^11^. Other work has enhanced the detection capability for small targets and subtle defects by substituting the traditional PAFPN in YOLOv8 with a Bidirectional Feature Pyramid Network (BiFPN) for efficient multi-scale feature fusion^12^. Further improvements based on YOLOv5 include the integration of an SA attention module—which combines channel and spatial attention—with grouping and channel shuffling techniques to reduce computation and improve detection efficiency^13^. Additionally, some researchers have replaced the original CIOU loss with an Inner-IOU loss function, which dynamically adjusts the scale of auxiliary bounding boxes to enhance regression accuracy and training speed^14,15^. Finally, binary classification methods from traditional machine learning have also been explored for steel defect detection, offering alternative perspectives for this field^16^.

#### YOLO model research

Current YOLO models typically suffer from two primary limitations: a large number of parameters and low accuracy in detecting small targets. These issues adversely affect overall detection performance and lead to high resource consumption. To address these challenges, several studies have proposed various solutions. For instance, one study replaced the original backbone with LHGNet to enhance feature extraction for small targets^17^. Another study employed the ShuffleNetv2 structure, which reduced the number of parameters while maintaining accuracy^18^. Additionally, some researchers utilized a multi-scale sequential feature fusion (SSFF) method to integrate features at different scales, thereby enhancing small target detection capability^19^. Other approaches have combined YOLOv4 with VTransformer for large-scale detection and classification to improve the model’s learning capacity^20,21^. Finally, some researchers incorporated the ELAN structure and CA module to consider both feature channel relationships and positional information in the feature space, ultimately improving detection accuracy^22^. Despite these advances, existing methods still involve a trade-off between lightweight design and small target detection performance, underscoring the urgent need for a novel model that achieves excellent small target detection while maintaining a low parameter count.

**Fig. 1:**
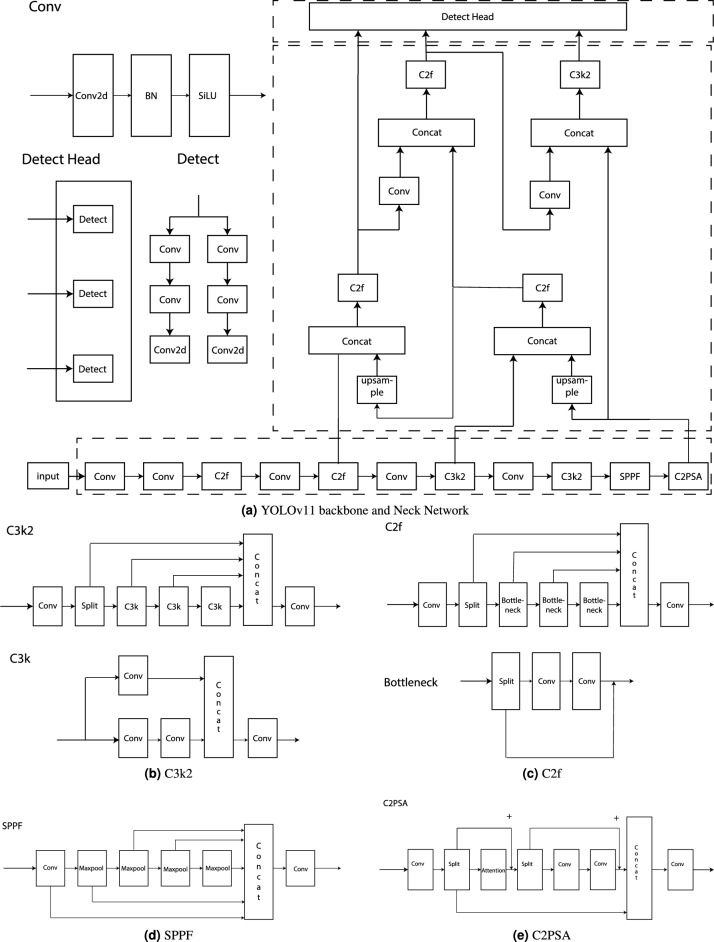
YOLOv11 network structure.

#### Feature fusion neck

This study employs YOLOv11n as the base network model (see Fig. 1). The architecture consists of four primary components: the input module, the feature extraction network, the feature fusion neck, and the output layer^23^. Prior to model input, the image data is processed using mosaic data augmentation, mixed image random cropping and splicing, adaptive corner calculation, and grayscale padding, and then scaled to the required training size.

The feature extraction network, which serves as the core of the model, utilizes CSPDarknet for target feature extraction and incorporates an SPPF module at its tail. The SPPF employs multi-dimensional pooling to convert inputs of arbitrary sizes into fixed feature dimensions, thereby enhancing the model’s ability to detect targets at various scales. In addition, the integration of the C2PSA module—which leverages spatial attention—enables YOLOv11n to focus on specific regions within the image, thereby improving accuracy and generalization in complex scenarios^24^.

The feature fusion neck comprises a feature pyramid network (FPN) and a path aggregation network (PAN) that perform top-down sequential feature fusion across multiple dimensions. This design enhances the model’s ability to predict target extents and improves its multi-scale prediction capability. Finally, the output layer produces the final prediction results from the detection head. Given the recurrent use of the Concat operation throughout this study, its functional diagram is presented in Fig. 4b

**Fig. 2:**
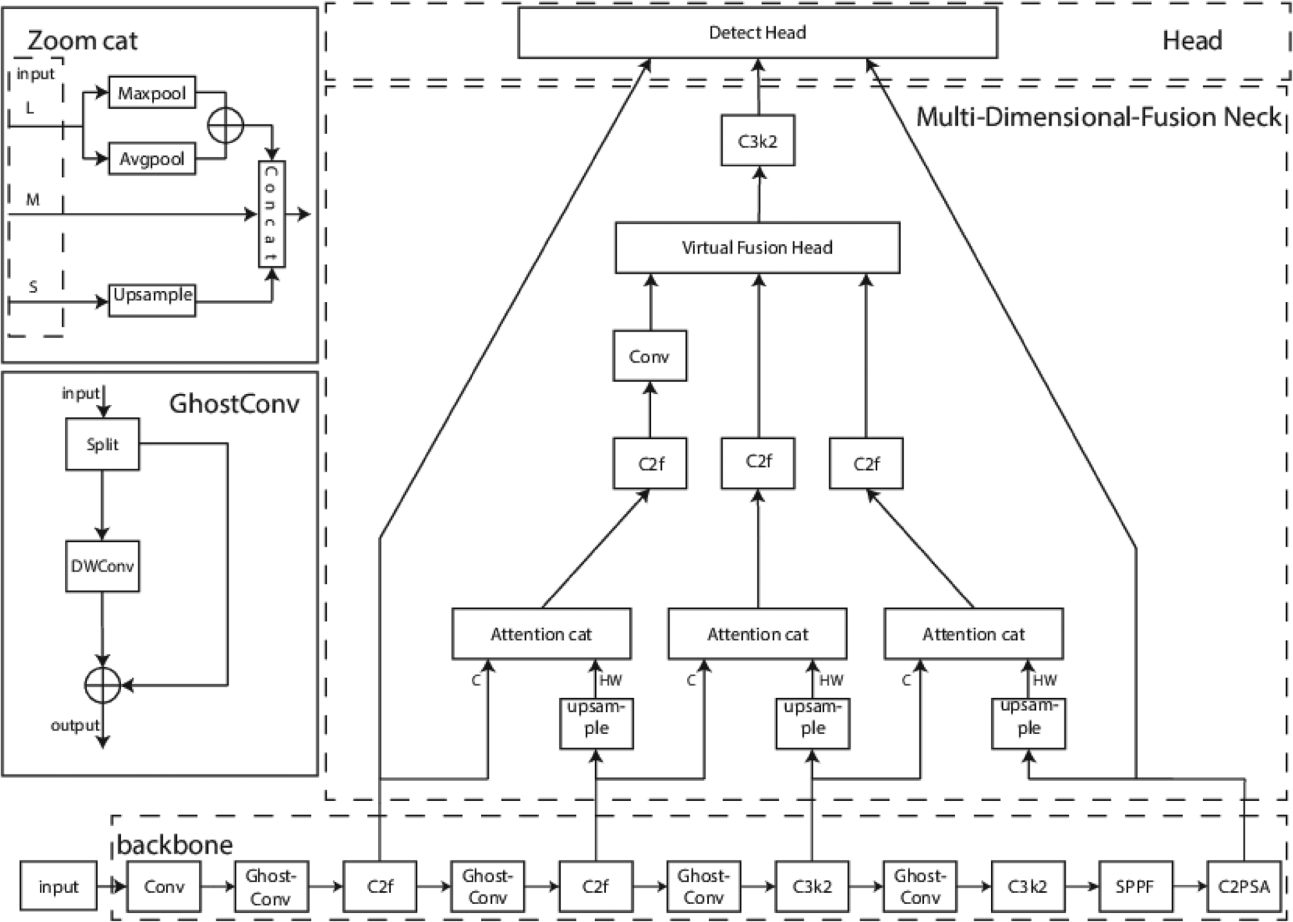
PSF-YOLO network structure.

#### Overview of the YOLO network neck structure

In recent years, to improve object detection performance in multi-scale, occluded, and small-target scenarios, researchers have extensively explored and continuously optimized feature pyramid structures, commonly referred to as “Neck” modules. The classic Feature Pyramid Network (FPN) effectively mitigates challenges related to scale variation by upsampling deep semantic features and fusing them with shallow detailed features.^25^ However, its unidirectional information flow limits the efficiency of feature interaction. To overcome this limitation, the Path Aggregation Network (PAN) introduces a bottom-up pathway atop FPN, forming a bidirectional, closed-loop information flow that facilitates reciprocal enhancement between shallow and deep features.^26^ This architecture has been widely adopted in mainstream models such as YOLOv4 and YOLOv5.

Subsequent improvements include Gold-YOLO, which proposes a Gather-Distribute (GD) mechanism that efficiently transmits multi-scale information, increasing inference speed by approximately 20% without sacrificing accuracy.^27^ ASF-YOLO integrates a dual-attention mechanism across both channel and spatial dimensions, dynamically focusing on target areas while suppressing background noise—resulting in a 3.1% improvement in small-target detection performance.^19^ SimNeck significantly reduces computational cost through design innovations such as parameter sharing, decoupled branches, and lightweight upsampling, achieving a 35% reduction in computation on the VOC dataset.^28^ To enhance robustness in occluded environments, CCFM incorporates a Transformer-based mechanism and cross-scale context modeling, effectively boosting detection accuracy.^29^

Among various Neck architectures, the Multi-scale Dense Fusion Neck (MDF-Neck) stands out for its use of dense connections to enable direct interaction across multiple scales. By incorporating skip connections, MDF-Neck preserves original information and facilitates feature reuse. Experimental results show that MDF-Neck improves mean Average Precision (mAP) from 1.5 to 2.0% on the custom GC10-DET+ benchmark dataset, demonstrating a strong balance between accuracy and structural efficiency. These advances mark MDF-Neck as a promising direction in the ongoing development of Neck design strategies.

### Improved YOLOv11 model

#### Overall design of the PSF-YOLO structure

The network architecture of the proposed PSF-YOLO model is illustrated in Fig. 2. In this design, the traditional Feature Pyramid Network (FPN) is replaced by a Multi-Dimensional-Fusion neck, which enhances the detection of small targets while optimizing the overall parameter count. Furthermore, the conventional DenseNet Concat operation is substituted with an Attention Concat that incorporates attention bias, rendering it more effective for feature fusion within the network^30^. Lastly, the standard convolutional layers in the feature extraction network are replaced with GhostConv layers, thereby reducing the number of parameters and simultaneously improving the model’s generalization capabilities. This integrated approach significantly enhances detection accuracy while maintaining a low parameter count.

#### Multi-dimensional-fusion neck

**Fig. 3:**
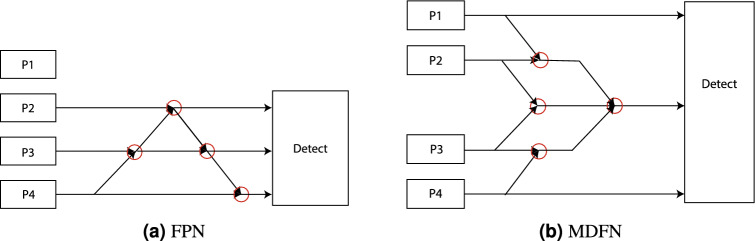
The fusion connection between MDFNeck and FPN.

Figure 3a illustrates the traditional YOLO feature fusion network. The conventional Feature Pyramid Network (FPN), a classic architecture in YOLOv11 , employs a backbone model originally designed for image classification and repeatedly integrates adjacent feature layers into a pyramid via top-down and lateral connections^31^. Other fusion networks, such as NAS-FPN, suffer from excessive parameter sizes, rendering them unsuitable for deployment on devices with limited storage capacity^32^.

In FPN, high-level feature layers are upsampled and fused with high-resolution, low-level feature layers to generate feature maps that retain both strong semantic information and fine spatial resolution. Although this structure is simple and efficient, it omits the P1 layer, resulting in suboptimal detection performance for small objects. Moreover, adding a P1 detection head introduces an additional input that significantly increases the model’s parameter size, as shown in Fig. 3b.

Our proposed neck structure overcomes these limitations by integrating the P1 layer while ensuring complete fusion of all feature maps. Specifically, every two adjacent feature layers are fused, and a virtual fusion head is employed to merge all layers into a single output containing information from the P1, P2, P3, and P4 layers. The resulting multi-dimensional feature maps are then further fused with the dominant feature layer, preserving diverse layer information and amplifying the influence of key features on the prediction results. Consequently, the model achieves enhanced small object detection performance while maintaining robust overall feature extraction capabilities, all within a balanced trade-off between parameter size and computational efficiency.

#### Virtual fusion head

The Virtual Fusion Head performs key operations including channel pooling, concatenation (Concat), and upsampling. When the inputs represent the large, medium, and small feature maps, respectively, the output Y is defined as follows:

1 $$\begin{aligned} Y=Connect(MaxPool(X_1)+Avgpool(X_1),X_2,Upsample(X_3)). \end{aligned}$$

Below, we describe the processing method for each feature map:

Small Feature Map: The dimensions of the small feature map are adjusted via upsampling so that its structure matches that of the medium feature map. Assuming the input tensor is in the forma $(N,C,H_{in},W_{in})$, , the output tensor becomes $(N,C,H_{out},W_{out})$, where

2 $$\begin{aligned} H_{out}=scale \times H_{in}, \end{aligned}$$

3 $$\begin{aligned} W_{out}=scale \times W_{in}. \end{aligned}$$

Here, the scale is a padding multiplier factor chosen based on the structure of the medium feature map to ensure that the final output format matches it. The padding algorithm and the filling value for blank regions can vary, with options including nearest, linear, bilinear, bicubic, and trilinear interpolation.

Large Feature Map: For the large feature map, channel pooling is applied. This method leverages both max pooling, which highlights the most salient features, and average pooling, which captures the overall characteristics of the input. This dual approach preserves key features of the original input, reduces the dimensionality for subsequent convolutional layers, compresses the model size, and improves processing speed. The resulting feature map is reformatted to match the structure of the medium feature map.

Medium Feature Map: The medium feature map serves as the reference format for all feature maps. Outputs from the small and large feature maps are adjusted to conform to the medium feature map’s dimensions. Finally, these feature maps are concatenated using the Concat operation, achieving an effective fusion of feature maps of different sizes.

#### Attention concat module

The Attention Concat module is designed to address the differing input dimensions of the feature maps on either side of the concatenation process, where the standard Concat operation tends to average feature propagation. To overcome this limitation, distinct attention mechanisms are applied to each branch, thereby emphasizing the extraction of complementary features from each input. Specifically, the first branch (Input1) focuses on spatial features by capturing interactions along the height and width dimensions, while the second branch (Input2) targets channel–spatial interactions, including both channel–width and channel–height relationships.

Traditional attention mechanisms, such as SENet^33^ and CBAM^34^, typically reduce dimensionality and introduce additional complexity during feature processing. In contrast, the triple attention mechanism performs residual operations directly on the original data without dimensionality reduction, thereby preserving the original feature structure, retaining richer contextual information, and enhancing feature propagation^35^. Building on this principle, the Attention Concat module emphasizes different aspects of the input data during feature concatenation.

The module employs the z-pool method—previously described in the Virtual Fusion Head module—which produces parallel outputs via max pooling and average pooling. In addition, the Channel Pool method is used to eliminate the channel dimension from the input data to the triple attention mechanism.

Equations (4), (5), and (6) describe the core computational steps of the Attention Concat module.Given two input feature maps $X_1,X_2\in R^{H\times W \times C}$, the final enhanced feature representation *Y* is generated as follows:

Equation (4) applies channel-wise attention recalibration:

4 $$\begin{aligned} X_{1}^{\text {out}} = X_1 \odot \text {CBS}(\text {ChannelPool}(X_1)), \end{aligned}$$

where

- *ChannelPool* A channel-wise pooling operation that aggregates spatial information (H$\times$W), producing a descriptor $s\in R^{1 \times 1 \times C}$.
- *CBS* A sequential module consisting of a $7 \times 7$ convolution, batch normalization, and sigmoid activation, yielding a channel weight vector $w_c \in [0,1]^C$.
- $\odot$ : Denotes channel-wise multiplication, recalibrating the original feature map $X_1$ with the learned weights $w_c$.

Equation (5) captures spatial context through a dual-branch attention design:

5 $$\begin{aligned} X_{2}^{\text {out}} = \text {Avg}\left( X_2 \odot \text {CBS}(\text {ZPool}(X_2^{(H,C,W)})), X_2 \odot \text {CBS}(\text {ZPool}(X_2^{(W,H,C)})) \right) \end{aligned}$$

- ZPool denotes dimension-permutation pooling:
  - First branch: Permutes the dimensions of $X_2$ to (*H*, *C*, *W*), then performs Z-pooling along the W-dimension, outputting $m_1 \in R^{W \times C \times 1}$.
  - Second branch: Permutes $X_2$ to (W, H, C), then performs pooling along the C-dimension, outputting m$m_2 \in R^{W \times H \times 1}$.
- **CBS**: Generates spatial attention maps $w_{s1}, w_{s2} \in [0,1]^{H \times W}$ using a $7 \times 7$ convolution.
- **Avg**: Performs element-wise averaging of the two weighted outputs to fuse spatial perspectives.

This structure enhances cross-dimensional feature interactions while maintaining computational efficiency through flexible tensor reorganization.

Finally, Eq. (6) concatenates the outputs from both attention branches:

6 $$\begin{aligned} Y = \text {Connect}(X_{1}^{\text {out}}, X_{2}^{\text {out}}), \end{aligned}$$

where:

*Connect*: Denotes concatenation along the channel dimension. This operation merges the strengths of channel attention and spatial attention, producing a unified, enriched feature representation.

**Fig. 4:**
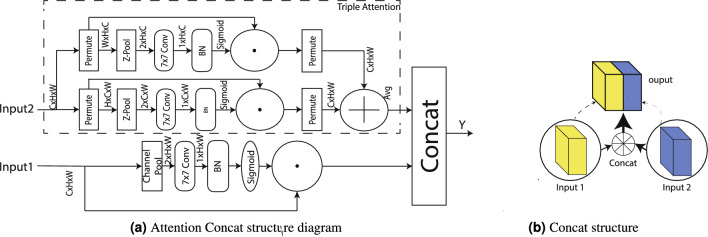
Attention concat and concat structure.

#### GhostConv module

In Fig. 2, the GhostConv section illustrates the structure of this module^36^. In our design, the traditional CBS module is replaced with Ghost Convolution(GhostConv). First, a standard convolution is applied to the input feature map to generate a set of intrinsic feature maps. Next, a grouped convolution is performed on these intrinsic feature maps to produce additional “Ghost” feature maps. Finally, the intrinsic and Ghost feature maps are concatenated to form the final output.

By generating Ghost feature maps and using simple linear operations to replace part of the conventional convolution computations, both the computational cost and parameter count are greatly reduced. Furthermore, the module decreases memory usage because the overhead associated with generating Ghost feature maps is low, while still maintaining a similar capacity for feature representation. This improvement in computational efficiency makes the module particularly suitable for devices with limited resources, as it can manage complex tasks without imposing a heavy computational burden. Most importantly, despite the significant reduction in computation, high accuracy is maintained.

We now quantify the reduction in parameter count achieved by replacing the conventional convolution module with GhostConv. The parameter gap is defined as follows:

7 $$\begin{aligned} & \text {Params}_{\text {gap}} = \text {Params}_{\text {Conv}} - \text {Params}_{\text {Ghost}} \nonumber \\ & \quad = k \times k \times C_{\text {in}} \times C_{\text {out}} - \frac{k \times k \times C_{\text {in}} \times C_{\text {out}}}{s} + \frac{d \times d \times C_{\text {out}}}{s \times (s-1)} \nonumber \\ & \quad = \frac{(s-1) \times C_{\text {out}} \times (k \times k \times C_{\text {in}} + d \times d)}{s}. \end{aligned}$$

Here, k denotes the convolution kernel size, d denotes the cheap kernel size, and s denotes the convolution stride.

## Experiment

### Experimental environment configuration

All experimental data in this paper were obtained using the following environment (Table 1):

**Table 1:** Configuration and training environment.

Environmental	Parameter value
CPU	12th Generation Intel Core i7 Processors
GPU	NVDIA GeForce RTX 3060 Laptop GPU
Memory	16GB
System	Windows 11 Home 24H2
CUDA	CUDA11.8
Programming language	Python3.9.19
Deeplearning framework	Pytorch2.0.1+cu118

### Dataset description

**Fig. 5:**
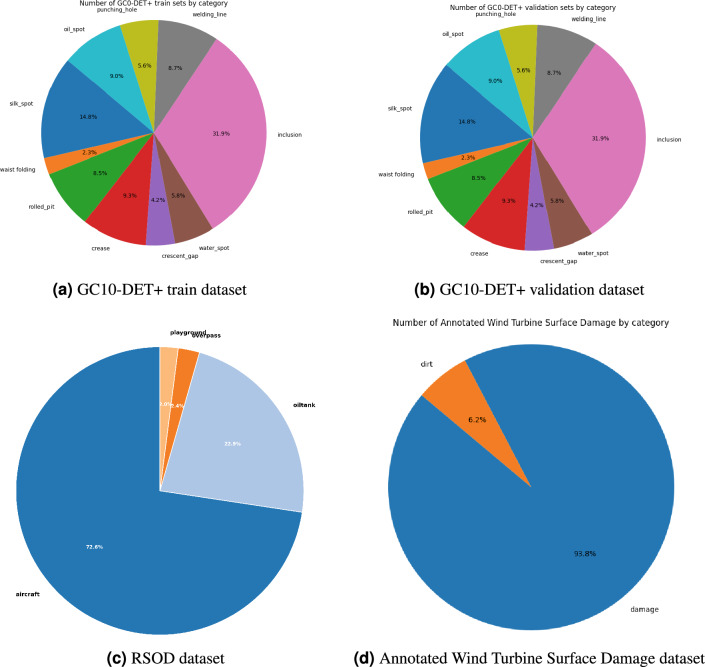
Dataset split ratio diagram.

The dataset used in this study, GC10-DET+, is primarily derived from the GC10-DET dataset^37^. Due to issues in public datasets—such as extremely low accuracy in certain categories and insufficient data volume—we addressed these shortcomings by sourcing additional images online, re-annotating them, and applying data augmentation through arithmetic transformations (vertical flipping, horizontal mirroring, and rotations by 90°, 180°, and 270°) to underrepresented categories. This process resulted in the enhanced GC10-DET+ dataset used in our experiments. To ensure optimal model fitting, the dataset was partitioned into training, validation, and test sets in a 7:1:2 ratio. The dataset comprises the following categories: Punching, Welding line, Crescent gap, Water spot, Oil spot, Silk spot, Inclusion, Rolled pit, Crease, and Waist folding. Figure 5a and b display the number of samples per category for the training and validation, respectively. To evaluate the generalizability of the proposed module, we conducted experiments on two publicly available datasets from distinct domains: RSOD^38,39^ and Annotated Wind Turbine Surface Damage datasets^40^. The RSOD dataset, designed for remote sensing applications, contains four categories: aircraft, oil tank, overpass, and playground. It was primarily used to validate the lightweight performance of our model.

In contrast, the V dataset focuses on turbine surface defect detection and comprises a single class, damage, which encompasses various types of risks encountered in diverse and complex scenarios. This dataset was utilized to further evaluate the model’s capability to detect small objects and to handle challenging, multi-scene environments.

Figure 5c and d show the label distributions for the RSOD and annotated wind turbine surface damage datasets, respectively.

### Explanation of evaluation metrics

Precision is defined as the proportion of correctly identified positive samples among all samples predicted as positive. It is expressed as the ratio of true positives (TP) to the sum of true positives and false positives (FP):

8 $$\begin{aligned} P=\frac{TP}{TP+FP}. \end{aligned}$$

Recall, which assesses the model’s ability to identify all relevant positive samples, is defined as the ratio of true positives to the sum of true positives and false negatives (FN):

9 $$\begin{aligned} R=\frac{TP}{TP+FN}. \end{aligned}$$

In deep learning, model performance is typically evaluated using precision and recall, which often exhibit an inverse relationship. To provide a more comprehensive assessment, the PASCAL evaluation framework employs Average Precision (AP). AP is derived from the precision-recall (P-R) curve, which plots precision against recall at different confidence thresholds, and is calculated by integrating precision over the recall range:

10 $$\begin{aligned} AP=\int _{0}^{1} PRdr, \end{aligned}$$

where p(r) is the precision at a given recall r. The mean Average Precision (mAP) is obtained by averaging the AP values across all n categories:

11 $$\begin{aligned} mAP=\frac{1}{n} \sum _{i=0}^{n}AP_i. \end{aligned}$$

Here, $AP_i$is the average precision for the ith category.

### Optimizer comparison analysis

Table 2 presents a comparative analysis of different optimizers on the GC10-DET+ dataset. The results indicate that SGD outperforms the other optimizers in terms of Recall, $mAP_{50}$, and $mAP_{50-95}$. Although its precision is not the highest, SGD demonstrates overall robust performance. Adamax follows closely; while its $mAP_{50}$ and $mAP_{50-95}$ scores are slightly lower than those of SGD, its precision is comparable, and it maintains a moderate recall. Adam achieves balanced performance across all evaluation metrics, with a relatively higher $mAP_{50}$. Nadam, AdamW, and RAdam exhibit similar results, particularly in $mAP_{50}$ and $mAP_{50-95}$; however, despite RAdam’s relatively high precision, its lower recall adversely affects its overall mAP performance. Both AdamW and Nadam offer stable performance in precision and recall, yet they do not surpass the performance of SGD or Adamax. In summary, SGD is the most suitable optimizer for this dataset, followed by Adamax, leading us to select SGD for our experiments.

**Table 2:** Performance comparison of model optimizers based on the GC10-DET+ dataset.

Optimizer	Precision	Recall	$mAP_{50}$	$mAP_{50-95}$
SGD	77.9%	72.7%	79.0%	42.5%
Adam	75.3%	71.3%	76.3%	37.9%
Adamax	77.4%	70.4%	78.1%	40.2%
AdamW	75.5%	70.4%	75.2%	40.0%
NAdam	75.5%	71.7%	75.7%	39.0%
RAdam	78.4%	67.4%	75.1%	38.2%

### MDF-neck

To ensure a fair comparison, we retain the same backbone network across all experiments and only replace the neck structures. Additionally, all models utilize three detection heads. Table 3 presents several commonly used neck architectures. It is evident that our proposed neck outperforms the alternatives in several key aspects.

In terms of parameter count, our model achieves a significant reduction—using 16.7% fewer parameters compared to the standard FPN neck. Regarding precision, our model reaches 77.6%. Although this is slightly lower than the FPN’s 77.9%, the difference is marginal. For recall, our model achieves 74.9%, the highest among all evaluated necks. The $mAP_{50}$ and $mAP_{50-95}$ are 81.0% and 43.9%, respectively. Compared with other neck models, our design leads by at least 1% in both metrics, indicating superior stability and robustness in object detection performance.

**Table 3:** Performance comparison of different necks.

Neck	Parameters	Precision	Recall	$mAP_{50}$	$mAP_{50-95}$
FPN	2,584,102	77.9%	72.7%	79.0%	42.5%
ASF-YOLO	2,509,501	76.9%	71.2%	76.1%	39.8%
Simneck	2,603,014	75.4%	75.1%	78.8%	40.4%
CCFM	2,174,550	80.1%	72.6%	80.1%	41.6%
Gold-YOLO	5,262,134	79.6%	73.8%	80.2%	42.0%
MDF-Neck	2,153,446	77.6%	74.9%	81.0%	43.9%

### Analysis of the attention concat module position

**Fig. 6:**
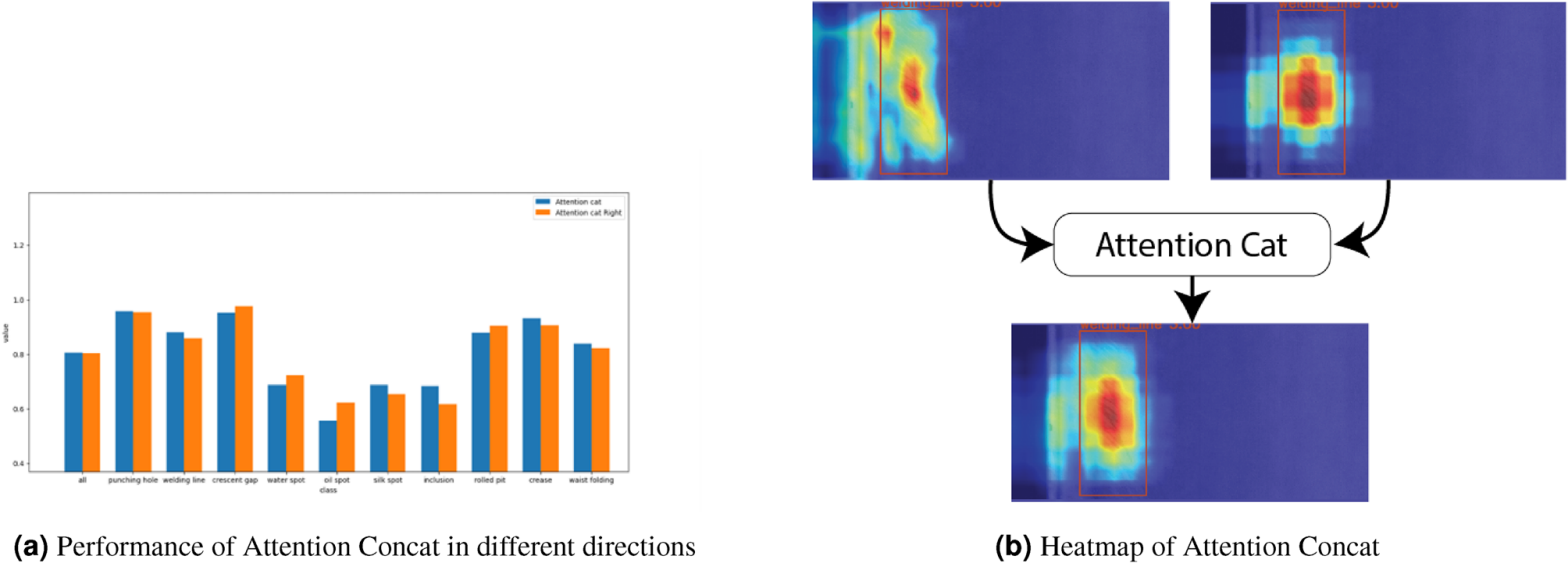
Performance comparison chart of “Attention Cat”.

Figure 6a clearly illustrates a significant difference in $mAP_{50}$ values between two configurations: “Attention cat” (with Input1 entering the deep layers and Input2 entering the low-dimensional layers) and “Attention cat Right” (with Input2 entering the deep layers and Input1 entering the low-dimensional layers). In most categories, “Attention cat” achieves higher $mAP_{50}$ scores than “Attention cat Right,” particularly in the punching hole, welding line, silk spot, inclusion, crease, and waist folding categories, where the differences are especially pronounced. Although “Attention cat Right” shows slightly higher performance in the crescent gap and rolled pit categories, these differences are minimal or occur in cases where precision is already very high. Overall, “Attention cat” demonstrates superior performance across a broader range of categories, which supports its adoption to enhance learning and adaptation for this task.

### Analysis of the attention concat module position

**Fig. 7:**
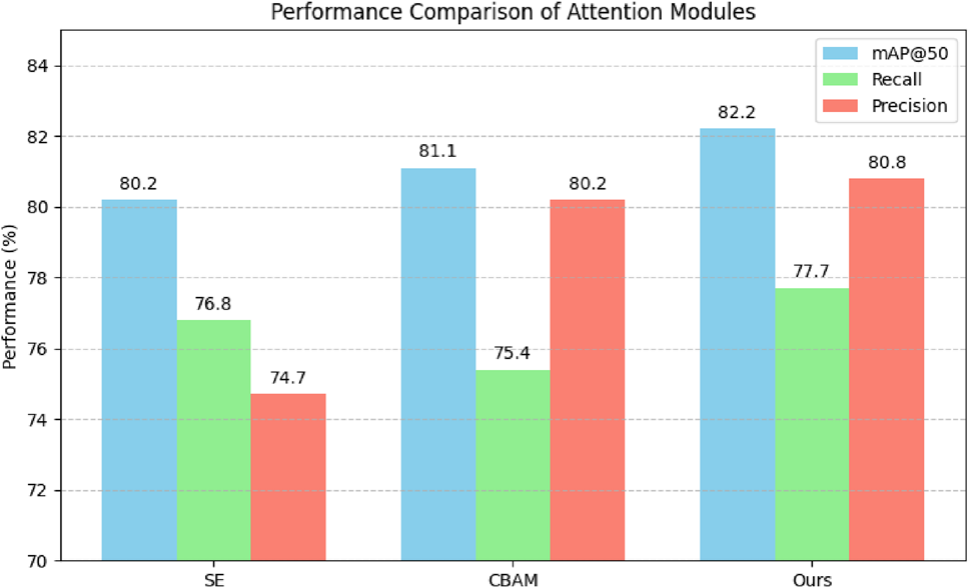
Performance comparison of attention modules.

From Eq. (5), it is evident that the triple-attention mechanism operates without explicit channel feature fusion. To evaluate the effectiveness of our proposed Attention Concat module, we replace the original triple-attention mechanism with two widely adopted alternatives: SE and CBAM. The experimental results, as illustrated in Fig. 7, show that our model consistently outperforms all comparative configurations. Specifically, it achieves the highest scores across all evaluation metrics: Precision (80.8%), $mAP_{50}$ (82.2%), and Recall (77.7%). These results highlight the superiority of the Attention Concat module in enhancing object detection performance.

### Comparison of different internal convolutions in the feature extraction network

Table 4 shows that GhostConv achieves the best overall performance by delivering high accuracy with a low parameter count, and it demonstrates strong results in both $mAP_{50}$ and $mAP_{50-95}$. This makes GhostConv particularly well suited for tasks requiring a balance between accuracy and efficiency.

In contrast, DWConv offers a favorable compromise between computational efficiency and performance, making it especially appropriate for resource-constrained scenarios, albeit with a slight reduction in recall. ResConv excels in high-precision object detection tasks, particularly in terms of $mAP_{50}$ and $mAP_{50-95}$; however, its higher parameter count imposes a greater computational burden, rendering it more suitable for applications on high-performance hardware.

Although both Conv and ODConv exhibit stable performance, their computational overhead is relatively high, and they are slightly less effective in terms of accuracy and recall compared to the other convolution types. Consequently, these methods are more appropriate for applications where computational resource constraints are not a critical concern.

**Table 4:** Comparison of different convolutions in feature extraction networks.

Convolution	Parameters	Precision	Recall	$mAP_{50}$	$mAP_{50-95}$
Conv	2,154,346	80.2%	75.1%	80.5%	43.4%
ODConv	2,174,814	79.7%	73.9%	80.2%	43.0%
GhostConv	1,918,426	80.8%	77.7%	82.2%	45.8%
DWConv	1,674,826	81.7%	74.8%	82.1%	44.1%
ResConv	2,369,866	78.3%	77.4%	82.6%	46.2%

### Ablation experiments

Table 5 summarizes the ablation study results. The original YOLOv11n network exhibits the lowest precision, recall, $mAP_{50}$, and $mAP_{50-95}$, while also having the highest parameter count and largest model size. Replacing the neck component leads to a comprehensive parameter optimization, with $mAP_{50}$ increasing to 81%. Subsequently, when the Concat operation and the convolution layers within the feature extraction network are replaced individually, a slight decrease in $mAP_{50}$ is observed. However, when all modules are simultaneously replaced—integrating modifications to both the Concat operation and the convolution components—the optimal model configuration is achieved. Overall, these improvements result in a significant reduction of 25% in parameter count, accompanied by a 3.2% increase in $mAP_{50}$ and a 3.3% increase in $mAP_{50-95}$.

**Fig. 8:**
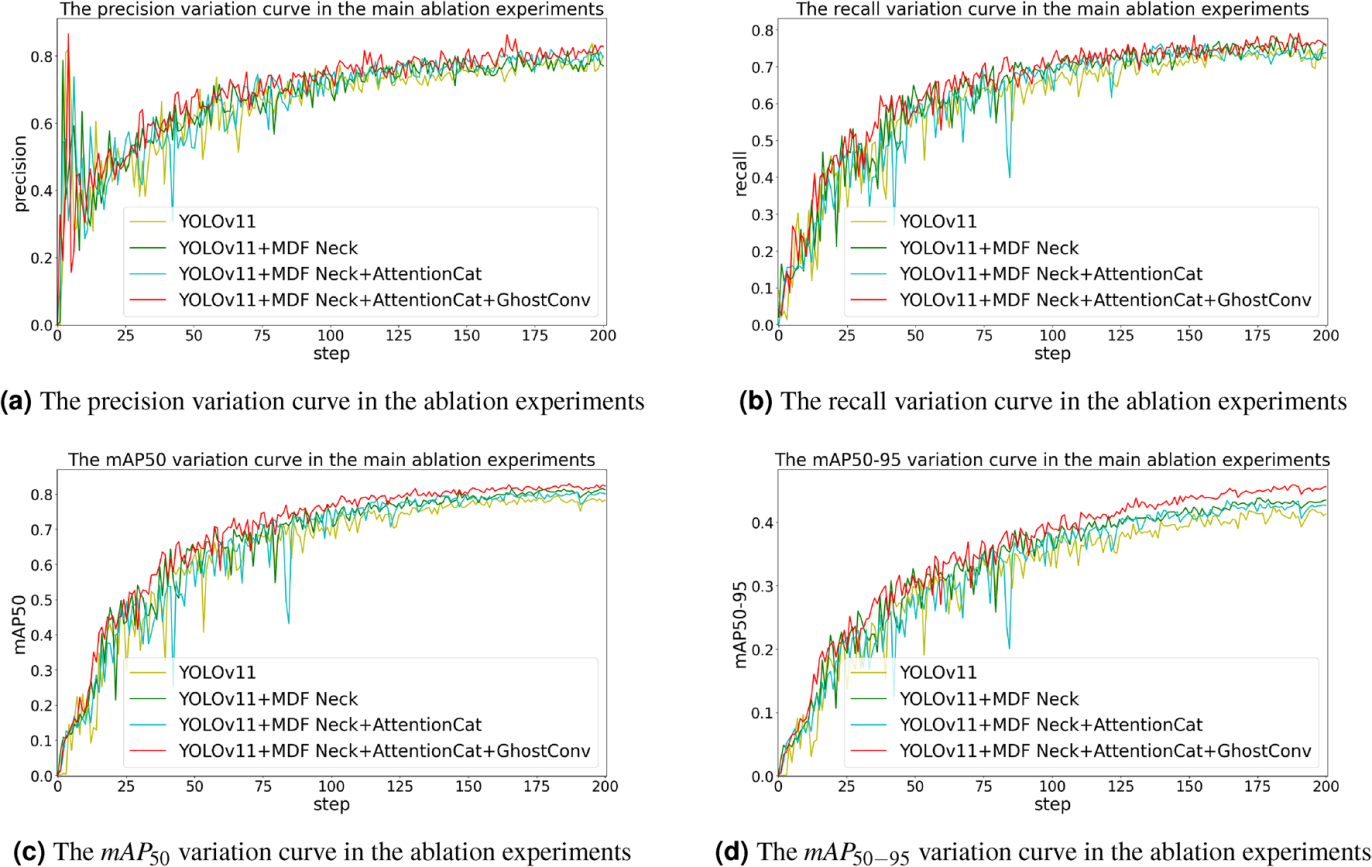
The training curves of $mAP_{50}$, $mAP_{50-95}$, Recall, and Precision across epochs for major module combinations.

Figure 8 presents the training curves of $mAP_{50}$, $mAP_{50-95}$, Recall, and Precision across epochs for major module combinations. Analysis reveals:

- Precision (Fig. 8a): Our model exhibits smaller oscillations and smoother convergence trends during the initial 20 epochs, ultimately achieving marginally higher precision than counterparts at convergence.
- Recall (Fig. 8b): Our model consistently maintains superior recall performance throughout training compared to other models.
- $mAP_{50}$ (Fig. 8c): Our model demonstrates notably smoother progression with minimal fluctuations, indicating more rational and efficient gradient descent compared to the significant oscillations observed in other models.
- $mAP_{50-95}$ (Fig. 8d): A clear performance advantage of our model over all alternatives is evident across the training trajectory.

**Table 5:** Ablation experiments.

MDF-Neck	Attention cat	GhostConv	Parameters	Precision	Recall	$mAP_{50}$	$mAP_{50-95}$	FPS
			2,584,102	77.9%	72.7%	79.0%	42.5%	122.87
		${\checkmark }$	2,348,182	81.8%	73.9%	80.0%	42.5%	136.44
	${\checkmark }$		2,585,302	82.4%	72.0%	79.2%	41.4%	131.95
	${\checkmark }$	${\checkmark }$	2,349,382	81.2%	74.5%	81.2%	43.6%	122.97
$\checkmark$			2,153,446	776.%	74.9%	81.0%	43.9%	101.74
$\checkmark$	$\checkmark$		2,154,346	80.2%	75.1%	80.5%	43.4%	86.14
$\checkmark$		$\checkmark$	1,917,526	82.2%	72.8%	80.4%	44.3%	116.21
$\checkmark$	$\checkmark$	$\checkmark$	1,918,426	80.8%	77.7%	82.2%	45.8%	116.34

### Detection effect analysis

**Fig. 9:**
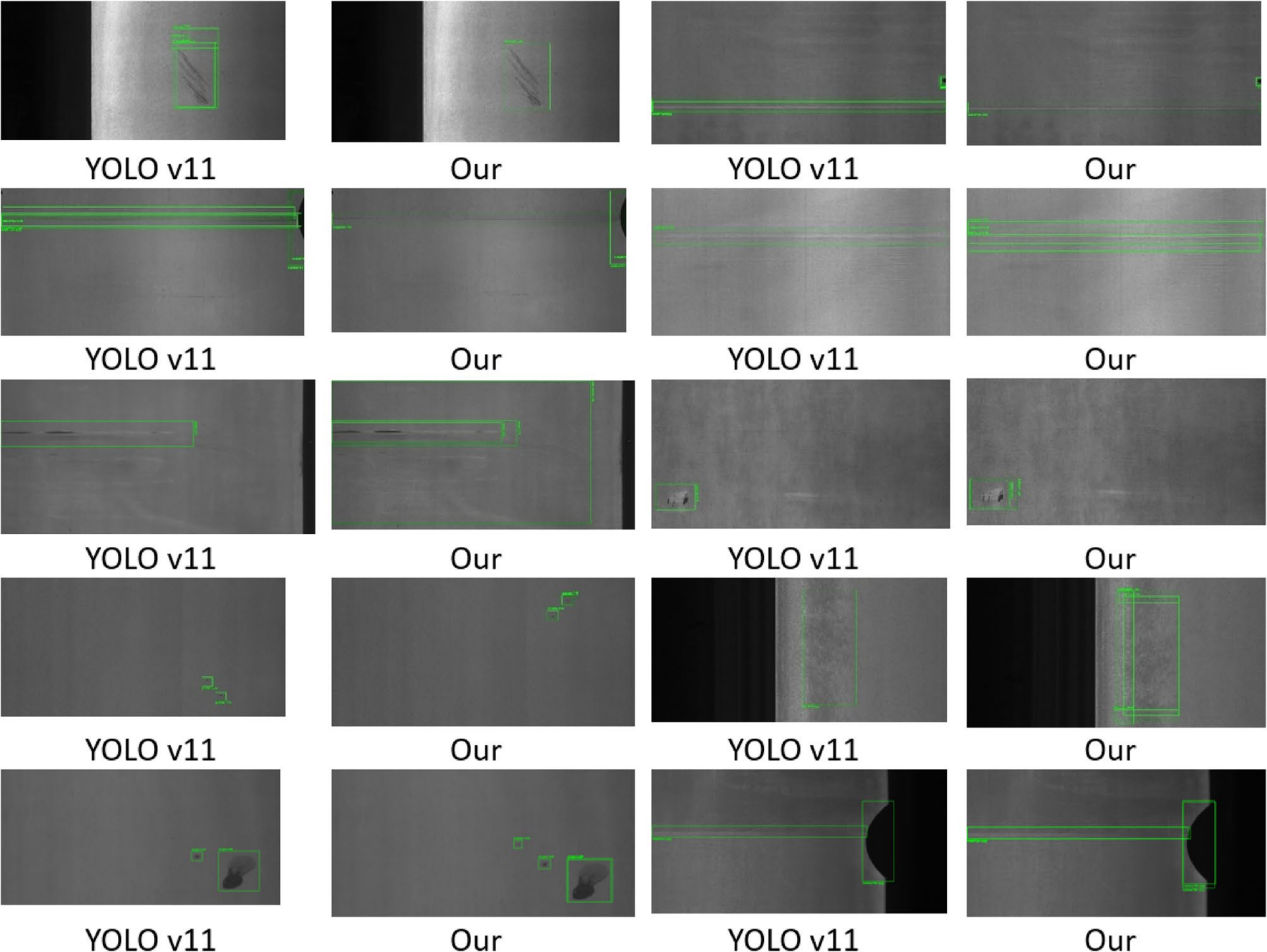
Performance test surface comparison chart.

Figure 9 presents several detection examples from our model on the test set. In terms of detection performance, the improved model demonstrates significant advancements in accuracy, robustness, and scene adaptability. Notably, the model exhibits higher confidence in all recognition tasks and a substantial reduction in misclassifications. Moreover, despite having a lower parameter count, the model achieves a marked improvement in recognition accuracy.

### Comparison with existing mainstream one-stage algorithms and improved backbones

Our analysis reveals that, compared to mainstream one-stage object detection algorithms, our model achieves the smallest parameter count. In comparison with an SSD model utilizing a VGG16 backbone, our model uses 249,457 fewer parameters and achieves a recognition accuracy that is 8.23% higher. Although our model’s mAP is slightly lower than those of YOLOv11n, YOLOv8n, and ASF-YOLO^19^ variants, it remains competitive. In particular, while our mAP is only 0.2% lower than that of YOLOv11s, the parameter count of YOLOv11s is five times higher than that of our model, underscoring the efficiency and lightweight nature of our design.

**Fig. 10:**
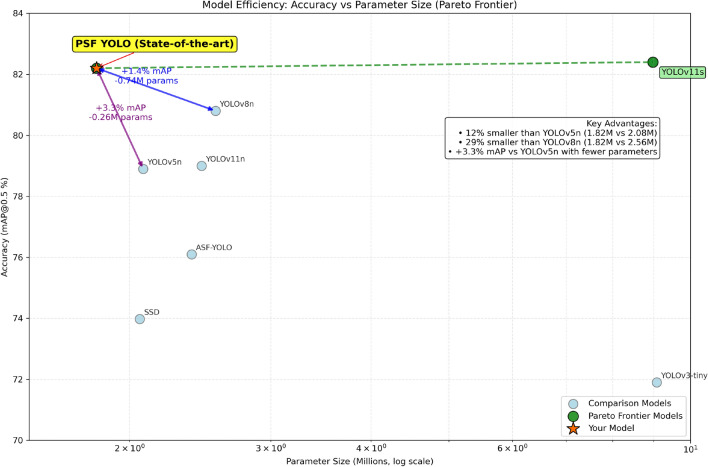
Model efficiency: accuracy vs parameter size (pareto frontier).

Figure 10 illustrates that the model demonstrates a Pareto advantage by achieving higher accuracy with fewer parameters. Specifically, it achieves a +3.3% improvement in $mAP_{50}$ over YOLOv5n while being 12% smaller in model size (1.82M vs. 2.08M), and 29% smaller than YOLOv8n (1.82M vs. 2.56M). These results highlight the model’s ability to optimize both performance and efficiency (Table 6).

**Table 6:** Comparison with the SOTA model on the GC10-DET+ dataset.

	Parameters	Recall	$mAP_{50}$	$mAP_{50-95}$	FPS
SSD	2,167,883	-	73.97%	-	-
ASF-YOLO	2,509,501	71.2%	76.1%	39.8%	171.3
YOLOv3-tiny	9,524,164	70.5%	71.9%	35.8%	269.58
YOLOv8n	2,686,318	73.8%	80.8%	44.3%	214.16
YOLOv5n	2,183,614	71.2%	78.9%	42.2%	222.08
YOLOv11s	9,416,670	74.5%	82.4%	46.8%	135.18
YOLOv11n	2,584,102	72.7%	79.0%	42.5%	122.87
Our	1,918,426	77.7%	82.2%	42.5%	116.34

### Experiments on the annotated wind turbine surface damage dataset and RSOD dataset

**Fig. 11:**
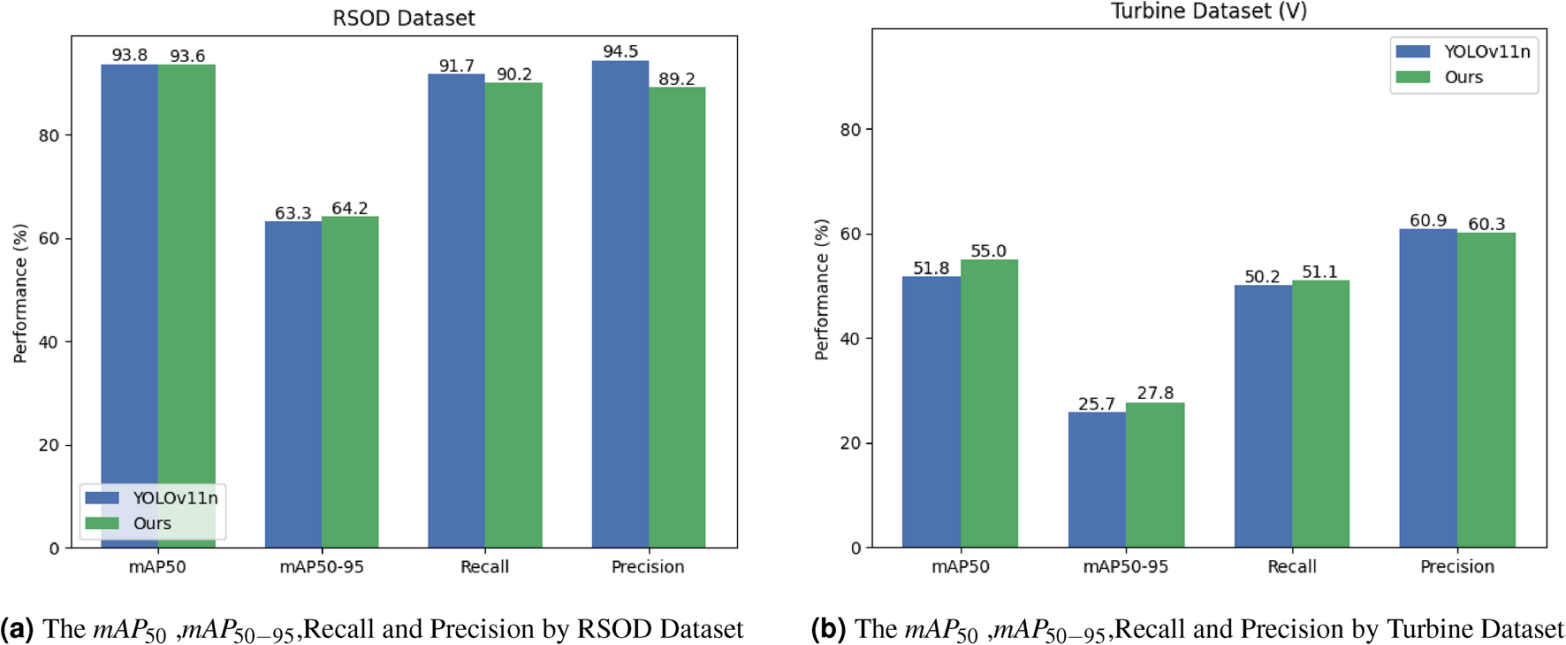
Annotated wind turbine surface damage dataset precision confidence curve.

To validate the model’s small-object detection capability under significant parameter reduction, we tested it on the aerospace remote sensing dataset RSOD. As shown in Fig. 11a and Table 8, experimental results demonstrate outstanding detection performance with a 25.8% reduction in model parameters: $mAP_{50}$ increased by 0.2 %, Recall increased by 1.5 %, and Precision increased by 5.3 %. Although $mAP_{50-95}$ slightly decreased by 0.9 %, this drop remains within the reasonable fluctuation range common in object detection tasks and fully meets practical application requirements. This conclusively proves the model effectively maintains small-object detection capability after lightweighting.

For the wind turbine surface defect dataset which presents challenges including high-resolution images, complex scenes, and difficult small-defect detection, we conducted robustness and generalization tests. As shown in Fig. 11b and Table 7, with a reasonable 25.8% parameter reduction, the model achieved comprehensive performance improvements: $mAP_{50}$ significantly increased by 3.2 % , $mAP_{50-95}$ increased by 2.1%, and Recall increased by 0.9 %. These results strongly demonstrate the model’s exceptional robustness and generalization capability when handling high-resolution, complex and variable scenarios.

**Table 7:** Comparison of models on an annotated wind turbine surface damage dataset.

Model	Parameters	Precision	Recall	$mAP_{50}$	$mAP_{50-95}$
YOLO v11	2,582,542	0.609	50.2%	51.8%	25.7%
Our	1,916,866	0.603	51.1%	55%	27.8%

**Table 8:** Performance comparison on the RSOD dataset.

Model	Parameters	Precision	Recall	$mAP_{50}$	$mAP_{50-95}$
YOLO v11	2,582,932	89.2%	90.2%	93.6%	64.2%
Our	1,917,256	94.5%	91.7%	93.8%	63.3%

## Conclusion

In this study, we propose PSF-YOLO, a lightweight and accurate object detection framework tailored for steel surface defect detection under resource-constrained industrial settings. To address the challenges of small defect sizes, complex backgrounds, and deployment limitations, we introduce a novel Multi-Dimensional-Fusion Neck (MDF-Neck) that integrates shallow-to-deep multi-scale features through adaptive weighting and dense connections.

We further incorporate a dual mechanism of Virtual Fusion Head and Attention Concat, which synergistically enhance feature aggregation and attention guidance, significantly improving the model’s sensitivity to subtle and low-contrast defects. In parallel, we systematically embed GhostConv into the backbone to reduce computational complexity and parameter count while maintaining high-level feature expressiveness.

Extensive experiments conducted on the improved GC10-DET+ dataset demonstrate that PSF-YOLO achieves a mAP@0.5 of 82.2% and a mAP@0.5 of 95% of 45.8%, surpassing multiple lightweight baseline models such as YOLOv11n and ASF-YOLO, with a 25% reduction in parameters. The model also shows robust generalization on a wind turbine blade defect dataset. These results validate the effectiveness, efficiency, and deployment potential of PSF-YOLO for real-world steel defect inspection scenarios.

## Data Availability

The data supporting the findings of this study are available from the corresponding author upon reasonable request and with explicit consent.
